# Radiographic Outcomes Following the Suture Fixation of Mid-pole Patellar Fractures

**DOI:** 10.7759/cureus.20448

**Published:** 2021-12-15

**Authors:** Bing Howe Lee, Michael Shen Xuanrong, Colin Wang Tzong-Yee, Yilun Huang, Keng Lin Francis Wong, Heng-An Lin, Merng-Koon Wong, Hamid Rahmatullah Bin Abd Razak

**Affiliations:** 1 Orthopaedic Surgery, Sengkang General Hospital, Singapore, SGP; 2 Musculoskeletal Sciences, Duke-National University of Singapore (NUS) Medical School, Singapore, SGP

**Keywords:** radiographic outcomes, union, tension band, suture fixation, patellar fracture

## Abstract

Background

Mid-pole patellar fractures are typically fixed with metal implants in the conventional “11-8” tension band construct. However, this technique is fraught with numerous implant-related complications. The aim of this study is to evaluate the union rate following “all-suture” fixation of mid-pole patellar fractures.

Methods

We retrospectively evaluated a consecutive case series of patients with displaced mid-pole patella fractures treated with “all-suture” fixation in our institution. Fifteen cases were available for this study. The average age was 61.5 years. Clinical and radiological outcomes were evaluated. Union time, complications, and revision rate were recorded. The minimum follow-up was one year.

Results

There were eight males and seven females, with a mean age of 61.5 ± 13.3 years. Fourteen out of 15 cases (93.3%) achieved radiographic union at 12 weeks postoperatively. The average time to radiographic union was 8.0 ± 2.7 weeks. Five cases (33.3%) had an increase in the fracture gap (>2 mm) at around four to six weeks postoperatively. Four of these cases had an eventual union, whereas one patient had fibrous non-union. There was one case of superficial surgical site infection and one case of infected hematoma. None of the patients required revision surgery.

Conclusion

“All-suture” fixation of mid-pole transverse patellar fractures is a safe and viable alternative to the conventional “11-8” tension band constructs with metal implants, with good union time, rates, and added benefits of not requiring additional surgery for implant removal.

## Introduction

Mid-pole patellar fractures are a relatively common injury and typically require surgical fixation [1,2]. Indications for surgery in mid-pole patellar fractures include intra-articular incongruity of more than 2 mm and loss of the extensor mechanism of the knee [3]. Currently, the surgical treatment of mid-pole patellar fractures involves various surgical techniques, most of which involve the use of metallic implants such as Kirschner (K-wires) and cerclage wires [2,4,5]. The most popular method is the Arbeitsgemeinschaft für Osteosynthesefragen (AO)-modified "11-8" tension band construct, which utilizes two longitudinal inter-fragment K-wires and a cerclage wire applied in a figure-of-8 fashion [6]. While the union rate of the "11-8" tension band constructs approaches 87.5%, numerous complications exist [7]. The metal implants are prone to breakage and implant prominence over time. Patients also often complain of pain secondary to soft tissue irritation from the prominent metal implants. Furthermore, there may be a need for a second surgical procedure for implant removal, increasing healthcare costs and risks to patients [8].

In our institution, we have adopted an "all-suture" technique to fix mid-pole patellar fractures. The benefits of this technique are that no metal implants are required, and hence there is no need for implant removal in the future. The aim of this study is to evaluate the union rate following the “all-suture” fixation of mid-pole patellar fractures at 12 weeks following surgery. We hypothesize that union rates following “all-suture” fixation of mid-pole patellar fractures are non-inferior to traditional “11-8” tension band constructs.

## Materials and methods

Ethics approval and subjects

SingHealth Centralised Institutional Review Board approved the conduct of this study (2020/2805). A retrospective review was conducted on all patients admitted to our institution from July 2018 to January 2020 with mid-pole patellar fractures and treated with suture fixation. Clinical data such as patient demographics, length of stay, and occurrence of postoperative complications were collected from the electronic health records.

Surgical technique

**Figure 1:**
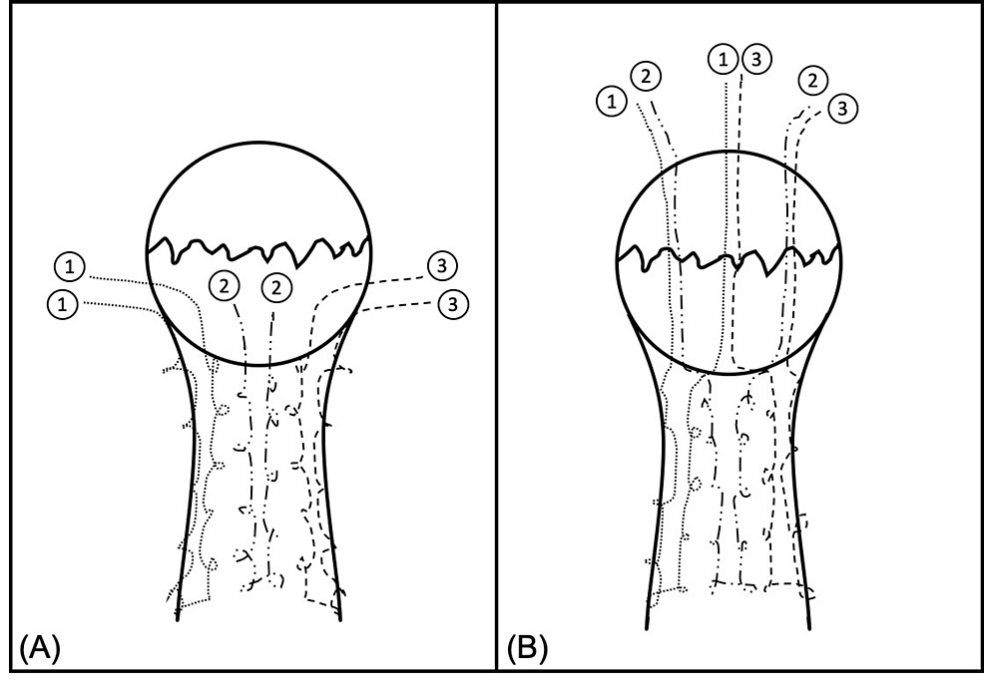
Illustration of suture technique (A) High-strength non-absorbable sutures are placed along the patellar tendon in Krackow locking fashion to form six cores. The suture systems are numerated 1–3 to show how they are cross-matched and pulled transosseously through the fractured patella with a 2.7-mm beath pin. (B) The cross-matched sutures are now at the superior pole of the patella after being pulled transosseously. The sutures are then "uncrossed" and tied like-to-like in sequence: suture 1 to suture 1, suture 2 to suture 2, and suture 3 to suture 3.

The principles of fracture surgery and the technique for suture fixation were similar in all patients. Patients were placed in a supine position on a radiolucent table with a pneumatic tourniquet placed over the thigh of the operated limb. A longitudinal midline skin incision was made, and dissection was taken down to the fracture site. Fracture hematoma and debris were cleared, and the joint was washed out. The fractured fragments were reduced, and the patella was held with a reduction clamp. If there was not already a rent in the patellar retinaculum, an incision was made either medially or laterally over the retinaculum to assess the articular congruity of the patella after reduction. The acceptable reduction was confirmed on fluoroscopy. High-strength non-absorbable sutures such as Ultrabraid® (Smith & Nephew, Memphis, TN, USA), FibreWire® (Arthrex, Naples, FL, USA), and/or No. 5 Ethibond® (Ethicon, Somerville, NJ, USA) were used to stitch the patella tendon from the inferior pole of the patella to the tibial tuberosity in a Krackow locking stitch fashion for a total of six cores (Figure 1).

**Figure 2:**
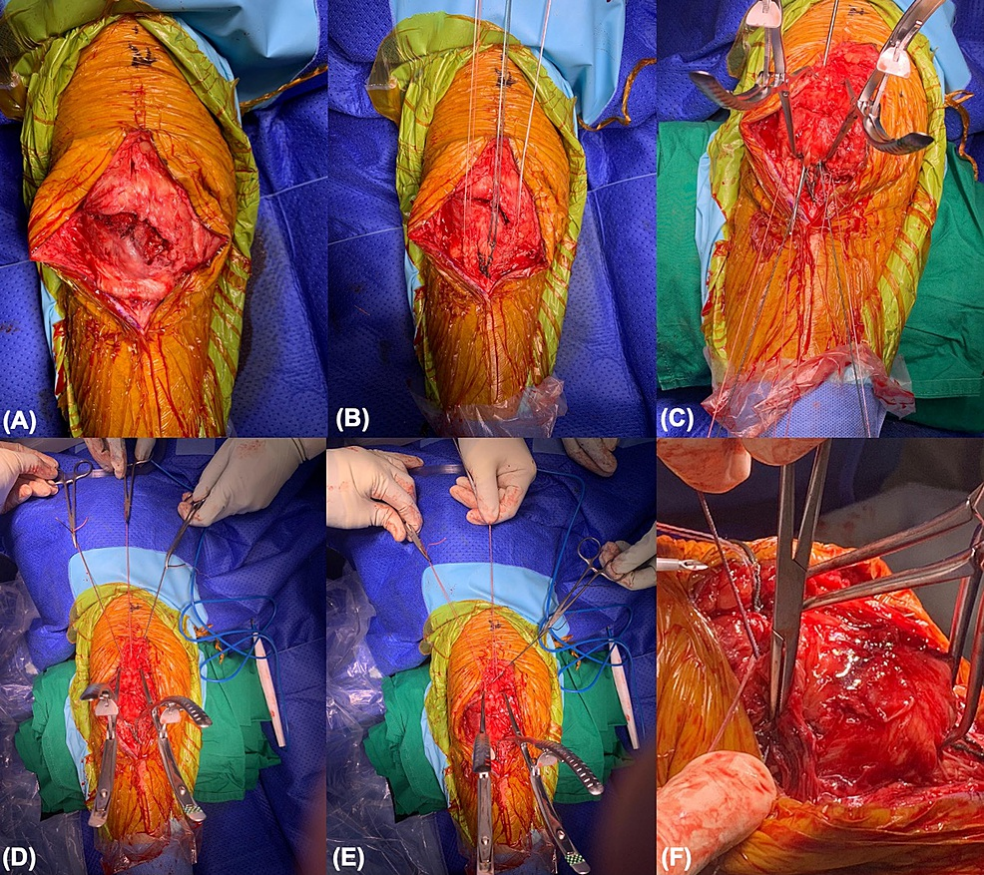
Intra-operative photos of the suture technique (A) A mid-pole transverse patellar fracture after dissection. (B) The patellar tendon is stitched with high-strength non-absorbable sutures in Krackow locking fashion to form six cores. (C) The suture systems are cross-matched, and a 2.7-mm beath pin is utilized to pull the sutures transosseously across the fractured patella. (D) The cross-matched sutures have been pulled through to the superior pole of the patella. (E) The suture systems are "uncrossed" and ready to be tied like-to-like. (F) A high-strength non-absorbable suture is used in a cerclage fashion and tied at the superolateral corner of the patella with a knot buried under the quadriceps tendon.

These sutures were then cross-matched and passed transosseously through the fracture site with a 2.7-mm beath pin, resulting in three parallel longitudinal tunnels of sutures. The sutures were then uncrossed and tied “like-to-like” on the anterior-superior corner of the patella. Suture knots were buried within the quadriceps tendon. In some cases, an additional non-absorbable suture was used as a "figure-of-8" cerclage suture. Fluoroscopy was used to confirm reduction. The closure was then performed in layers with Prolene 3/0 to the skin. Figure 2 shows the surgical technique in a step-by-step fashion.

A few pearls for the surgical technique shared by the authors are given in Table 1.

**Table 1:** Pearls of surgical technique

No.	Tips
1.	Ensure good reduction prior to passing sutures through.
2.	The transosseous tunnel should be as narrow as possible to allow suture passage to avoid "play of sutures" within the tunnel.
3.	Before tying the sutures “like-to-like,” ensure that they are pulled taut and uncrossed.
4.	Make stab incisions in the quadriceps tendon before tying the sutures to ensure that they are buried and the knots are as close to the patella bone as possible.
5.	Consider an additional non-absorbable suture as a "figure-of-8" cerclage.
6.	There may be a role for self-tightening suture materials such as DYNACORD, but further studies are required.

Postoperative protocol

Patients were placed on a ranging knee brace postoperatively and were allowed to bear weight as tolerated and ambulate with the brace from the first postoperative day. Patients were taught to avoid active quadriceps contraction for the first two weeks. Upon discharge, patients were reviewed in the outpatient clinic setting at two weekly intervals with serial radiographs taken to assess the maintenance of reduction and eventual fracture healing. Progression of range of motion was dependent on the fracture configuration as well as radiographic findings (i.e., ROM 0-30 degrees for two weeks; 0-60 from three to four weeks; 0-90 from four to six weeks; beyond 90 degrees after six weeks titrated to patient and fracture factors). Patients were gradually progressed to the full range of motion of the knee in the span of three months following the fracture fixation.

Radiographic assessment

X-ray radiographs were taken on day 1 and 2 weekly thereafter until 12 weeks postoperatively or when radiographic union was achieved. Radiographic union was defined as the presence of cortical and trabecular bridging and disappearance of the fracture line on the anterior-posterior and lateral radiographs of the knee [9,10]. The presence of fracture displacement was reviewed on the lateral radiograph. A significant fracture displacement was determined as more than 2 mm. Two independent reviewers (L.B.H and H.R.B.A.R) analyzed the radiographs to improve inter-observer reliability. The radiographic union rate was calculated by dividing the number of patients with complete fracture healing at 12 weeks following surgery by the total number of patients.

## Results

A total of 15 consecutive cases were available for review. There were eight males and seven females, with an average age of 61.5 ± 13.3 years. Five had fixation of the right patella, and 10 had fixation of the left patella. The minimum follow-up was one year.

**Figure 3:**
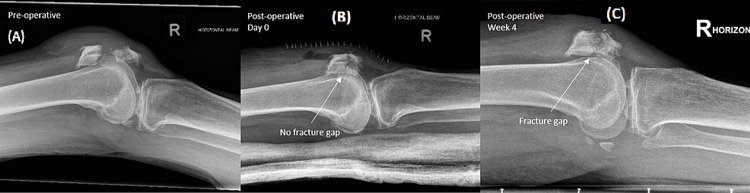
Radiograph of a mid-pole transverse patella fracture fixed with suture technique (A) Preoperative x-ray showing the mid-pole displaced transverse patella fracture. (B) Immediate postoperative x-ray after suture fixation of the patella fracture. (C) Four weeks postoperative x-ray showing fracture gap displacement of around 2 mm.

The average range of motion of the knee was 0 to 115 (± 19.5) degrees at one year. Fourteen out of 15 cases (93.3%) achieved radiographic union at 12 weeks postoperatively. The average time to radiographic union was 8.0 ± 2.7 weeks. One-third (five) of the cases had an increase in the fracture gap (>2 mm) at around four to six weeks postoperatively. An example is shown in Figure 3.

Four of these cases had an eventual union, whereas one patient had fibrous non-union and further displacement of the fracture fragments. This patient was last reviewed at one year postoperatively and had clinical union without any functional limitations.

One patient had persistent fever postoperatively and was reviewed by the infectious disease specialist for postoperative-infected hematoma that required six weeks of antibiotics. He recovered unremarkably without the need for any surgical drainage or implant removal. There was a case of superficial skin infection, which resolved with oral antibiotics. None of the patients complained of suture prominence or required any revision surgery one year postoperatively.

A summary of the clinical data of the 15 study patients included in this study is presented in Table 2.

**Table 2:** Clinical data

S. No	Age (years)	Gender	Side	Fracture union	Time to union (week)	Fracture displacement	Post-op complications	Revision surgery
1	43	Male	Left	Yes	4	No	No	No
2	61	Female	Right	Yes	8	Yes	No	No
3	59	Female	Right	Yes	6	No	No	No
4	38	Male	Left	Yes	6	No	No	No
5	54	Male	Right	Yes	6	No	No	No
6	61	Female	Left	Yes	10	Yes	No	No
7	78	Male	Left	Yes	6	No	No	No
8	69	Male	Left	Yes	12	No	No	No
9	73	Female	Right	Yes	8	No	No	No
10	65	Male	Left	Yes	8	No	Infected hematoma	No
11	41	Male	Left	Yes	12	No	Superficial surgical site infection	No
12	66	Female	Right	Yes	6	Yes	No	No
13	86	Male	Left	No	Non-union	Yes	Fibrous non-union	No
14	63	Female	Left	Yes	6	No	No	No
15	66	Female	Left	Yes	12	Yes	No	No

## Discussion

The present study shows that the “all-suture” fixation technique is a safe and viable alternative to the conventional “11-8” tension band constructs, with good union time and rates.

Traditionally, the tension band wiring technique has been the preferred choice of procedure for the treatment of displaced mid-pole patellar fractures. The AO technique involves two longitudinal Kirschner wires and an 18-gauge stainless steel wire in a figure-of-eight pattern looped over the anterior patella [6]. However, stainless steel can be a difficult material to manipulate through tissues, and this may result in a poor fixation [11]. Moreover, this surgical technique is fraught with implant-related complications such as migration or breakage of the Kirschner wires and prominent wire knots. This results in up to 60% of patients complaining of symptomatic hardware, necessitating a second surgery for the implant removal [12-14]. This increases healthcare costs and risks to the patients [8]. For these reasons, alternative solutions of patellar fracture fixation have been reported in the literature to reduce the complications related to the use of metallic implants [15-21].

In our institution, we adopted an “all-suture” technique for the fixation of mid-pole patellar fractures. High-strength non-absorbable braided polyester sutures are passed through transosseous tunnels in a parallel longitudinal fashion to secure the fracture fixation and, if required, are further reinforced with a figure-of-eight cerclage suture. Studies in the literature have supported the use of such non-absorbable braided polyester sutures, suggesting that their high tensile strength is comparative to metal wires [16,17,19,21]. A biomechanical cadaveric study by Patel et al. comparing transverse mid-pole patellar fracture fixation using metal wires versus braided polyester sutures demonstrated that the quality of fixation with braided polyester sutures was comparable to that of stainless steel wires [22]. They also found that braided polyester sutures provide sufficient stability to withstand loads encountered during postoperative rehabilitation. Wright et al. also concluded that FiberWire® was more resistant than steel in a biomechanical study of transverse patellar fractures [23]. An in-vitro study by Gerber et al. has also demonstrated the high tensile strength and stiffness of braided polyester sutures compared to other non-absorbable or absorbable sutures in the repair of rotator cuff tendons [24]. This material also has minimal tissue reactions and is safe for clinical use [20,25]. Evidence in the literature has reported good outcomes with the use of a variety of suture materials (FiberWire®, Ultrabraid®, Ticron® [Covidien, Dublin, Ireland], and Ethibond®). These sutures were placed following different configurations such as circumferential fixations, transosseous osteosynthesis, and figure-of-8 configurations. A systemic review by Camarda et al. concluded that suture fixation is able to deliver good clinical outcomes and reduce the rate of surgical complications and re-operation [17]. In this study, Ultrabraid®, FibreWire®, and Ethibond® sutures were used. To date, there is no study in the literature that has compared these three types of sutures and their tensile strength in patellar fracture fixation. However, it is evident that the use of a non-absorbable suture fixation for patellar fractures is a viable substitute for metal implants.

In the present study, there was a 93.3% radiographic union rate at 12 weeks postoperatively. The average time to radiographic union was 8.0 ± 2.7 weeks. This is comparable with the reported union rates and time of suture fixations in the literature. In a matched control study by Chen et al. comparing 25 patients with displaced patellar fractures who underwent transosseous suturing versus 25 patients who had tension band wiring fixation, the union time was 8.43 ± 2.92 for the suture group and 8.62 ± 2.82 for the tension band group [16]. There was no statistical difference between both groups with regard to union time. The union rate of patellar fractures fixed with metal implants ranges from 87.5% to 97.3% [7,26]. This is comparable to the reported 93.3% union rate in our study. This suggests that the union time of patellar fractures fixed with sutures is non-inferior to those fixed with the metal constructs.

In this present study, we had five patients who had an increase in the fracture gap, noted at four to six weeks postoperatively. Four of these cases went into an eventual union, while one had fibrous non-union radiographically at 12 weeks. This phenomenon of fracture gapping in suture fixation has been previously reported by Camarda et al. They also noted a loss of reduction (<4 mm) in two patients at four weeks postoperatively before eventual union [21]. This increase in fracture gap can possibly be explained by suture loosening after soft tissue atrophy [20]. The resultant instability can potentially result in further fracture displacement and non-union. Chen et al. recommended routinely splitting the quadriceps tendon in the anterior-superior corner of the patella to eliminate soft tissue interposition between sutures and the bone to achieve secure fixation [16]. A biomechanical study by Heygen comparing a new self-tightening suture material DYNACORD™ with FibreWire® in the repair of rotator cuff tendons in sheep has shown promising results [27]. A future biomechanical evaluation of the efficacy of the use of self-tightening sutures in patellar fracture fixation would be beneficial.

For the patient with fibrous non-union, he was an elderly 87-year-old gentleman with osteoporosis on antiresorptive therapy. Fracture displacement coupled with poor bone quality and healing could be the underlying factors. This patient did not require any revision surgery as he was able to ambulate without pain and had no functional limitations. In this series, no patients required implant removal. This finding is consistent with other studies in the literature. In a systemic review by Camarda et al. on non-metallic implants for patellar fractures, they noted that only four patients (3.2%) required additional surgery for implant removal. This is in contrast to the reported 40%-61% rate of hardware removal in patellar fractures fixed with metal implants [8,28]. Reul et al. studied 113 surgically treated patellar fractures and concluded that extensive tension band constructs were the main determinant of poor outcome and increased economic burden due to higher re-intervention rates. They also suggested that surgeons should consider less onerous implants [8].

We also did not experience any cases of prominent suture knots, and we recommend burying the suture knots within the quadriceps tendon on the anterior-superior surface of the patella after securing the suture knots. This has been suggested by other authors [11,16,20]. We also had two cases of postoperative infection, with one patient requiring an extended duration of antibiotics for infected post-op hematoma. This patient recovered unremarkably without the need for any implant removal. In a comparison study by Gosal et al. on 21 patellar fractures treated with “11-8” tension band wire construct versus 16 patellar fractures fixed with suture, they reported a higher infection rate in the former, which has significant implications on patient morbidity [19]. Bacterial adherence and biofilm formation is a well-known sequela of implant infections in orthopedic procedures. This often necessitates implant removal for adequate and effective treatment of the infection [29]. Studies have looked into the development of an antibiotic coating on surgical sutures as a means to reduce post-implantation infections [30]. Future studies could evaluate the efficacy of these antibiotic-coated sutures in “high infection risk” patients with patellar fractures.

One of the strengths of this study is the inclusion of a homogenous sample of only transverse mid-pole patellar fractures. This is in contrast to other studies that also included superior and/or inferior pole fractures. Our surgical technique and postoperative rehabilitation protocol were also consistent in all patients. This study has several limitations. As this was a retrospective analysis, there are the usual issues of the potential inaccuracy of medical records and information bias. The relatively small sample size may have also potentially weakened the power of analysis. Moreover, although all patients improved functionally with no long-term complications at 12 weeks postoperative, we did not collect any validated functional scores. Moving on from this case series, we are looking to conduct a more robust prospective cohort study to further evaluate the outcomes following suture repair of mid-pole patellar fractures.

## Conclusions

“All-suture” fixation of mid-pole transverse patellar fractures is a safe and viable alternative to the conventional “11-8” tension band constructs with metal implants, with good union time and rates and an added benefit of not requiring additional surgery for implant removal. We noticed an increase in the fracture gap at four to six weeks postoperatively in 33% of patients. This is likely due to suture loosening after soft tissue atrophy. A future biomechanical evaluation of the efficacy of the self-tightening suture's use in patellar fracture fixation would be beneficial.
